# The changing landscape of text mining: a review of approaches for ecology and evolution

**DOI:** 10.1098/rspb.2024.0423

**Published:** 2024-07-31

**Authors:** Maxwell J. Farrell, Nicolas Le Guillarme, Liam Brierley, Bronwen Hunter, Daan Scheepens, Anna Willoughby, Andrew Yates, Nicole Mideo

**Affiliations:** ^1^ Department of Ecology & Evolutionary Biology, University of Toronto, Toronto, Ontario, Canada; ^2^ School of Biodiversity, One Health & Veterinary Medicine, University of Glasgow, Glasgow, UK; ^3^ MRC-University of Glasgow Centre for Virus Research, Glasgow, UK; ^4^ Université Grenoble Alpes, CNRS, LECA, Laboratoire d'Ecologie Alpine, Grenoble, France; ^5^ Department of Health Data Science, University of Liverpool, Liverpool, UK; ^6^ School of Life Sciences, University of Sussex, Brighton, UK; ^7^ Division of Biosciences, University College London, London, UK; ^8^ Odum School of Ecology, University of Georgia, GA, USA; ^9^ Informatics Institute, University of Amsterdam, Amsterdam, The Netherlands

**Keywords:** Natural Language Processing, large language models, deep learning, literature synthesis, Information Extraction, database construction

## Abstract

In ecology and evolutionary biology, the synthesis and modelling of data from published literature are commonly used to generate insights and test theories across systems. However, the tasks of searching, screening, and extracting data from literature are often arduous. Researchers may manually process hundreds to thousands of articles for systematic reviews, meta-analyses, and compiling synthetic datasets. As relevant articles expand to tens or hundreds of thousands, computer-based approaches can increase the efficiency, transparency and reproducibility of literature-based research. Methods available for text mining are rapidly changing owing to developments in machine learning-based language models. We review the growing landscape of approaches, mapping them onto three broad paradigms (frequency-based approaches, traditional Natural Language Processing and deep learning-based language models). This serves as an entry point to learn foundational and cutting-edge concepts, vocabularies, and methods to foster integration of these tools into ecological and evolutionary research. We cover approaches for modelling ecological texts, generating training data, developing custom models and interacting with large language models and discuss challenges and possible solutions to implementing these methods in ecology and evolution.

## 1. An overview of approaches

Tools from linguistics, computer science, and machine learning can aid in all phases of text-based research, such as identifying relevant papers, analysing research trends, constructing or expanding databases, and building automated pipelines to translate text into data ready for statistical analysis. Despite the power and utility of text mining, it has yet to be adopted widely in ecology and evolution [1]. Farrell *et al*. [1] present recent uses in ecology and evolution, and discuss future applications, but a major gap remains for life science scientists to learn about available methods and their underlying philosophies. Here, we review the growing landscape of approaches and methodological paradigms for text mining. To build a vocabulary necessary for collaboration with linguists and computer scientists, throughout the review, we define key terms and concepts (see table 1 for definitions of underlined terms). Our aim is to encourage further uptake of text mining by detailing how individual steps can be tailored to goals in ecology and evolution, and combined within an overall pipeline akin to other analytical fields [2]. We begin by describing the evolution of text mining tools and map them to three broad ‘paradigms’: (i) frequency-based approaches, (ii) traditional Natural Language Processing (NLP), and (iii) deep learning. As recent advances in NLP have turned to deep learning-based models, we highlight approaches to train models specific to ecology and evolution, and make the most of the rich training data in existing ecological databases, and practices for interacting with language models for extraction and synthesis of data in ecology and evolutionary biology.

### (a) Paradigm 1—quantifying text: frequency-based approaches

One of the most common outputs of text analysis is the word cloud, a hallmark of literature reviews, scientific talks, and personal websites of academics. This method takes a segment of text and identifies individual words, counting how often each appears, and displays them with font size proportional to frequency. A word cloud is an example of wider ‘bag-of-words’ approaches, a paradigm based on quantifying frequencies of words within and/or across documents. Here, the meaning and order of words are ignored, instead treating them as discrete units, much like quantifying the abundances of species in a given location.

When considering words or phrases (collectively, terms) independent of their context, such as in a bag-of-words analysis, ‘stop words’ are often filtered out. Stop words are common, uninformative words that provide no specific meaning or context (e.g. ‘the’, ‘are’, ‘we’, ‘why’). Lists of stop words are included in NLP software libraries [3,4], but there is no single definitive list and choice of words to remove, or whether to filter words at all will depend on the task and nature of the texts to be analysed. Rather than filtering specific words, researchers may opt for a threshold approach by removing X% of the most and least common terms across documents. These choices reinforce that bag-of-words approaches do not capture the meanings of words, their order, or their contexts. In linguistics, word sense refers to the multiple meanings a word can take depending on its context. For example, ‘bark’ could refer to the outermost layer of a woody plant or the sound dogs make. Without the context, a bag-of-words approach would consider these terms equivalent. Rather than using single words as terms of interest, further context can be captured in a frequency-based approach by focusing on groups of ‘*n*’ words at a time in shifting windows, termed n-grams, (e.g. the phrase ‘text mining’ is a 2-gram). How one chooses to go from raw text to distinct terms will change their number and frequency, ultimately influencing downstream analyses and interpretation.

Beyond word clouds, exploratory analyses of single terms or n-grams commonly involve quantifying the similarity of documents through document-term matrices (akin to site-by-species matrices in community ecology). Terms can be weighted by how frequently they occur in any document, for example by using a term-frequency-inverse document frequency (TF-IDF) weighting, which takes into account the frequency of terms within and between documents. This captures the importance of a term to a document, adjusted for the fact that some words appear more frequently across the whole set of documents. TF-IDF matrices can be used for many downstream tasks including text summarization, keyword identification, and as a way to transform text and use it as a predictor or an input to downstream models. For example, ‘topic modelling’ is an unsupervised approach that uses a method like latent Dirichlet allocation to group documents into abstract topics that can be further explored. Nunez‐Mir *et al*. [5] review the ecological applications of topic models and other similar approaches to identify hidden themes in a body of literature and demonstrate their utility for systematic and exploratory literature reviews. Bag-of-words approaches can also be useful in text classification. For example, instead of unsupervised methods like topic modelling, labels can be attached to documents and used to develop models for information retrieval (e.g. systems that identify ‘relevant’ documents). For example, naive Bayes classifiers based on word frequencies can achieve >90% accuracy in some scenarios, such as identifying advertisements relevant to the wildlife trade [6].

### (b) Paradigm 2—structural insight: traditional NLP

While word frequencies can help to explore the content of texts and be used in document classification, the order and wider context of words impart additional meaning, which can be key to understanding texts. However, enabling computers to understand human language, with all of its variations and irregular, evolving forms and rules is a complicated challenge. Computational techniques for the analysis of human language, a discipline called Natural Language Processing (NLP), typically involves a series of algorithms, each using linguistic rules to perform a discrete task and identify patterns in text.

**Figure 1.**
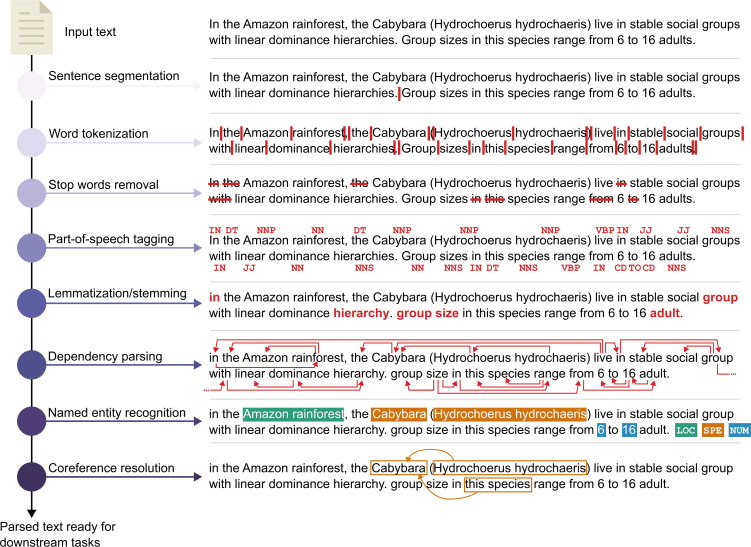
Illustration of common text processing tasks, including sentence segmentation, stop words removal, tokenization, POS tagging, lemmatization/stemming, dependency parsing, named entity recognition and coreference resolution. POS tagging involves marking up tokens with a set of descriptive POS tags, e.g. determiner (DT), proper noun (NNP), adjective (JJ), etc. Dependency parsing creates a tree-like representation of the grammatical relationships between words in a sentence. Note that the order and inclusion of individual steps in a pipeline will depend on the task. Example text derived from Herrera *et al*. [7].

An NLP pipeline (figure 1) starts with a set of texts of interest, known as a corpus (singular) or corpora (plural). This raw text is often referred to as unstructured data, as opposed to data in a table or other format ready for analysis. Many pipelines start with some form of tokenization, which splits text into smaller units, referred to as tokens. This might be paragraphs into sentences, or phrases into individual words, punctuation, or words into sub-words. One specific case of tokenization is sentence segmentation which aims to isolate individual sentences. This can be as simple as splitting at every period or can employ more complex algorithms [8,9]. In languages without compound words, such as English, word tokenization can be as simple as separating whenever there is a space [3], but becomes more difficult in languages such as German or Mandarin where spacing may not be present between individual words. Note that sentence segmentation and tokenization can be used as preliminary steps before bag-of-words approaches, but we include them here as tokenizers are often designed using a mix of linguistic and computational considerations [10]. The ultimate goal of tokenization is to capture units of text that have a discrete meaning for the given task, and thus may include rules for different punctuation marks, or algorithms that collapse multiple words into a single token. For example, the Penn Treebank [11] tokenizer uses regular expressions to tokenize individual sentences and is therefore able to split common English contractions (e.g. ‘don’t’ becomes ‘do’ and ‘n’t’).

Once tokens are identified, we can tag each word based on its part of speech (POS) (noun, verb, adjective, adverb, etc.) by using POS taggers. POS tagging can be achieved through different approaches including rule-based, probabilistic, or hybrids of the two [12]. Having tagged parts of speech can make additional pre-processing easier by helping identify multiple forms of words sharing a base form. For example, if one wants to identify instances of infectious disease, their interest likely covers all variants (or surface forms) of the word ‘infect’: infects, infecting, infection, infectious, infectivity, etc., and treating these words as independent may skew frequency-based analyses. Stemming can mitigate this by removing end characters from words to collapse down to a given base form, or stem (in the above example, ‘infectious’ becomes ‘infect’). However, stemming can often confound superficially similar yet unrelated words (e.g. *universal, university*, *universe* would all be stemmed to *univers*). Lemmatization considers a language’s full vocabulary, as well as the surrounding context of a word and its POS, to collapse a word down to its canonical form, or lemma. For example, ‘infectious’ again becomes ‘infect’ but ‘better’ becomes ‘good’, a relationship not captured by simple stemming. Once stemmed or lemmatized, stop word removal can also be considered (see Paradigm 1). For simple models, the choices made in lemmatizing and stop word removal can have a large influence on model outcomes [13].

Dependency parsing is another processing step enhanced by POS tags. These methods analyse grammatical structure to tag relationships between different words in a sentence. This allows for many downstream tasks such as identifying the subjects of sentences, who is doing what action, or where and when events are occurring. Similarly, POS tags can help when words are spelt the same but have different meanings depending on the context (e.g. words used as either a verb or a noun: ‘bark’, ‘breed’, ‘control’, ‘flower’), or sets of words that represent a concept or a formal group. Sets of one or more words that comprise a single concept or entity can often be identified through named entity recognition (NER) (also known as entity identification, entity chunking or entity extraction) which is the process of identifying objects or concepts of a certain type, such as people, locations, events, etc. For example, rather than the more common use as a verb, the word ‘swallow’ could be recognized as an ‘organism’ entity in the phrase ‘most species of swallow feed on insects’ based on the context of use. Developing NER tools typically requires these entities to be labelled, from which a model can be developed to predict these entities.

In biomedical text analysis, specialized NER tools have been developed to identify genes, proteins [14], and other terms used in medical language [15]. In ecology and evolution, NER has largely focused on identifying taxonomic names [16–20], though there are many other potential uses [1]. Once entities are recognized, coreference resolution aims to identify all the terms in a given text that refer to a particular entity. For example, NER identifies the occurrence of a named entity (e.g., ‘animal’), but does not recognize when two different entities refer to the same thing (e.g. ‘swallow’ and ‘*Hirundinidae*’). Coreference resolution includes finding and resolving different types of references, such as words that refer back to the subject (anaphora/cataphora), coreferring noun phrases across sentences, etc.

Beyond NER, there are myriad possibilities for tailoring NLP pipelines to work with text from specific fields. Entity linking is a method of disambiguation that assigns a unique identity to entities in a text and allows researchers to cross-reference entities with external databases or knowledge systems across diverse disciplines [21–28]. Note that coreference resolution does not necessarily use an external reference, whereas entity linking does. Once entities have been correctly identified and resolved, one may want to extract semantic relationships between entities (e.g. predator–prey, gene–protein). Similar to entity linking, relation extraction (RE) is a core task in the field of Information Extraction (IE). By leveraging dependency and entity recognition as above, researchers can transform unstructured information in texts into structured relational or tabular data [29], ready for further analysis. Although RE is a complex task that can be done through many approaches [30], it will be an essential task for automating the creation, expansion, and maintenance of biodiversity datasets as the scientific literature grows [1].

### (c) Paradigm 3—language models: deep learning in NLP

In general terms, a language model is a method that predicts the next word in a sequence, given the previous words. There are two main types of language models: n-gram models and artificial neural network based models. N-gram models predict words from a fixed window of previous words, with probabilities estimated by counting the frequencies of these n-grams in a corpus [29]. N-gram language models can be simple and efficient, but suffer from poor generalization to words and word sequences not seen in the training data—by predicting the next words using only the words that immediately precede, n-gram models ignore useful information from more distant words in a text [31]. Neural language models are more complex and computationally expensive, but offer many unique advantages owing to their ability to learn vector representations from the text itself. The process of self-supervised learning generates supervisory signals from the data itself, for example by masking some part of the input data and having the model try to predict it. Self-supervised learning enables large unlabelled corpora to be used for training models and is now commonly used to train large language models that are then fine-tuned to perform more specific tasks through additional training. In this way, neural language models can learn complex features from unstructured data and capture subtle patterns which may be challenging for traditional NLP models.

Instead of treating words simply as indices in a vocabulary, neural network models represent words as vectors of numbers (also called word embeddings, a form of vector representation). A word embedding is a real-valued vector representation of a word that encodes meaning and preserves semantic proximity among words. While bag-of-words approaches like the TF-IDF can be used as numeric representations of text, neural embeddings are learned by iterating over a corpus of text and learning the association between the words. By assuming that terms with similar words before and after are semantically similar, embedding models learn vector representations of the words and their average contexts. In this way, distances between words in a continuous vector (measured by cosine similarity for example), indicate how similar the words are expected to be in their linguistic meaning (i.e. how replaceable one word might be with another).

NLP has seen a revolution starting with the incorporation of neural network based models in the 2010s, and with the development of new ways to represent text [32] that work well with deep learning methods [33,34]. Deep learning is now ubiquitous in NLP research, often superseding traditional NLP pipelines [35]. Various deep neural network (DNN) architectures have been used to address NLP tasks including convolutional neural networks (CNN), recurrent neural networks—most famously represented by the long-short term memory (LSTM) and the bi-directional LSTM models—and graph neural networks [36]. Similar to first-generation neural language models (word2vec, GloVe), these DNN-based models aim to produce meaningful word embeddings. The deep learning model that sparked the ‘NLP revolution’ is the Bidirectional Encoder Representations from Transformers (BERT). BERT’s main novelty is the use of a transformer [34] as a neural architecture for encoding context in word embeddings [33]. Transformers are a family of encoder–decoder neural networks that use the mechanism of self-attention to capture long-range dependencies between distant elements in a sequence. In the context of NLP, self-attention enables transformers to learn the context of a word relative to the entire input sequence. This contrasts with earlier approaches, including static embeddings (e.g. word2vec [32]), which represent a word as the same vector regardless of its context, and directional models (e.g. LSTM [37]), which only capture sequential dependencies (left-to-right and/or right-to-left). For example, word2vec will have the same embedding for the *bark* of a tree and the *bark* of a dog, while a transformer will look at the surrounding words to generate an embedding that captures the meaning of ‘bark’ based on the context in which it occurs. Unlike word2vec, which learns a single embedding per word, BERT breaks down words into sub-words, allowing it to model embeddings for individual characters all the way through to complete words [10]. By combining sub-word embeddings, a BERT model can generate vector representations of out-of-vocabulary words, increasing its utility across different corpora and domains.

Following BERT’s breakthrough, many alternative neural language models (e.g. XLNet, RoBERTa, ELECTRA) have been developed that use different architectures, hyperparameter tuning, pre-training objectives, and additional training data [38–40]. Since the pre-training of these language models does not require labelled data, there has been a trend of training increasingly large models using more and more massive unlabelled corpora. These large language models (LLMs) push computational boundaries through a mix of new neural architectures and sheer increases in size (number of parameters, size of vocabulary, number of learned vectors, all reflecting a large amount of pre-training literature) [35,41,42]. LLMs pre-trained on vast amounts of text often serve as foundation models that can be adapted to a wide range of downstream tasks through additional supervised learning with smaller datasets [43](see section 2b - *Transfer learning: adapting existing language models*). Foundation models are typically very large, with billions of parameters, and the past few years have seen rapid growth in the size of LLMs [35]. Such massive language models are computationally and financially expensive to train [41,44]. Several lines of work aim to create smaller language models with lower computational requirements but comparable performance [45–47] (e.g. ALBERT, DistilBERT, MobileBERT, TinyBERT, LLaMA and, Mixtral). As the environmental impact of training and using large language models is not negligible, this should be considered when developing new NLP models [48].

Most large language models are pre-trained on general-domain corpora. For instance, the first iteration of BERT was trained on English Wikipedia (2500M words) and the BookCorpus (800M words) [49]. Although general-domain LLMs pre-training corpora are derived from multiple massive sources, they may not be diverse enough to generalize to a specific domain or task that involves a specialized vocabulary, e.g. ecology and evolutionary biology. Pre-training to a specific domain can lead to increased performance in both high and low resource settings [50]. Some models have been pre-trained using scientific corpora such as SciBERT, trained on a random sample of 1.4 million papers from Semantic Scholar [51] [52], BioBERT [53], a biomedical language model, expands on BERT using additional training data from PubMed abstracts and full-text articles, and PubMedBERT is trained on 14 million abstracts from PubMed [54]. These models have become the launch points for custom NLP pipelines for scientific and biomedical text [14,55], allowing for advances in building literature-based biomedical databases. Despite their demonstrated use in biomedical sciences, large language models are just beginning to be adopted in ecology and evolution [1,16,56–58], and to our knowledge, there is currently only one large language model, BiodivBERT, trained explicitly on biodiversity-related texts [56].

## 2. Using language models in ecology and evolution

**Figure 2.**
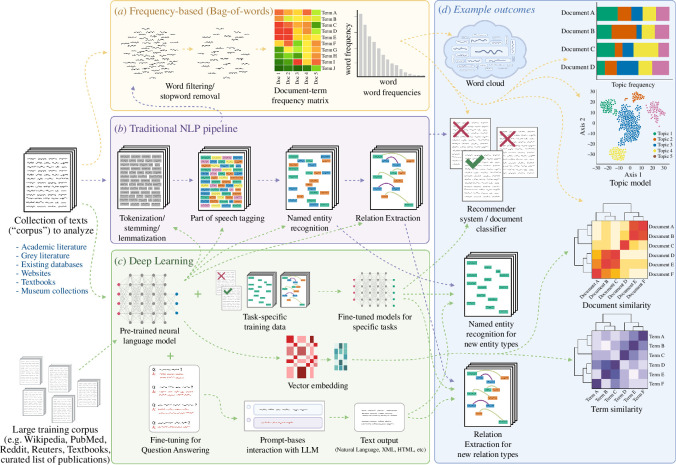
Example steps for three text mining paradigms: (*a*) frequency-based (bag-of-words) (frequency-based) approach, (*b*) traditional NLP pipeline, and (*c*) deep learning-based language models. Dashed arrows indicate possible interactions between each of the paradigms (e.g. text pre-processed using a classical NLP pipeline could be analysed using bag-of-words approaches, or fed into a deep learning-based document classifier). (*d*) Some examples of outcomes, including quantifying document similarity, topic modelling and training models for document classification, named entity recognition and relation extraction. Note that for steps in the traditional NLP section, there are often additional task-specific external data sources (e.g. word lists, dictionaries, labelled training data) which are not depicted here.

Text mining is a broad domain with an array of approaches that can be used for the same tasks. Above we outlined three broad paradigms (frequency-based, traditional NLP, and deep learning). While deep learning-based language models are revolutionary and an extremely active area of research, methods and tools from each of the three paradigms can be effective for analysing text in ecology and evolution, even if not considered the current ‘state of the art’. Often simpler rule-based tools can be more computationally efficient, and perform better for a specific task than a more general, probabilistic machine learning-based approach. Furthermore, the three paradigms can interface with each other. For example, rule-based methods can be used for generating labelled data for supervised training of machine learning models. More generally, the output of one type of task can be used as inputs to the next (figure 2). For example, Kulkarni & Di Minin [59] and Hunter *et al*. [60] use TF-IDF to find and remove duplicate articles, then use a hashing function to turn texts into features for use in a neural network-based classification model. However, as large language models are likely to provide the greatest flexibility and potential for future development, we focus the rest of the article on their application, highlighting challenges and solutions to developing ecology-specific language models, gathering suitable training data, and identifying avenues for prompt-based interaction with LLMs.

### (a) State of the art in ecology and evolution: learning in a low-resource domain

Despite many promising applications of NLP in ecology [1,19], we currently have few domain-specific NLP tools [16,56]. This is in part because ecology is a low-resource domain [61], meaning there are few large open-access text databases available for training foundation models [1], and there are few gold standard databases with task-specific labels needed for supervised learning. Some ecology-specific gold standard datasets exist, such as COPIOUS, a corpus directed towards the extraction of species occurrences from the biodiversity literature [62], and the newer BiodivNERE [63], a gold standard corpus for NER and RE. While gold standard datasets are essential for comparing the performance of new approaches as they are developed, they do not currently provide enough examples to train deep learning NLP models from scratch. A major barrier to text mining in ecology and evolution is the lack of data formatted for the training, testing, and development of domain-specific NLP tools. Even with a concerted effort, it is unlikely that we will reach the same quantities of annotated data that are available for other deep learning-based ecological tasks such as computer vision to identify species in camera traps [64,65].

Gathering labelled training data for language models typically involves manual tagging of documents, phrases, or words by expert annotators. Given the increasing availability of computing power, expert labelling and curation of training data can be the most expensive and time-consuming component of a custom NLP model. The cost is often high because of the manual effort of (potentially highly trained) humans. When tasks are difficult or require domain expertise, there may be trade-offs between the annotators’ time and number of examples needed for training, the need for multiple annotators to limit individual biases and provide a consensus annotation, or crowdsourcing annotations to produce training sets at the risk of poorer quality or less reliability among annotators. As an alternative to manual curation, we can use a mixture of simple rules based on the content of existing databases to increase the efficiency of labelling [66]. In ecology and evolution, comparative databases of species traits, biodiversity responses and experimental data built from primary literature [67,68] offer excellent resources for creating labelled training data. These are typically manually constructed by multiple contributors, have undergone some quality checks,and represent decades of effort curating facts and observations from scientific literature.

When biodiversity databases can be paired with source texts, approaches such as data augmentation and weak supervision can be used to efficiently generate labelled data [61]. Data augmentation involves artificially expanding a dataset by applying transformations to existing labelled data. Widely used in computer vision, transformations which preserve the semantics of the images, such as image rotation, flipping or cropping, are applied to a dataset to construct additional synthetic data. This technique has only recently been adopted in NLP because the nature of text makes it more difficult to derive transformations that maintain the semantics of the original sentences, however, new techniques for data augmentation in NLP are being developed [69,70]. Unlike data augmentation, which requires an initial set of labelled data, weak supervision uses unlabelled texts for which labels are obtained (semi-)automatically from simpler rule-based methods (e.g. outputs of Paradigms 1 and 2) or pattern matching based on external information sources [66]. A popular form of weak supervision is distant supervision, in which labels are derived from existing knowledge bases, dictionaries, and other forms of structured knowledge sources [71]. The main challenge with weak supervision is reducing the noise and inaccuracies introduced by these imperfect annotation sources.

### (b) Transfer learning: adapting existing language models

If only a small set of labelled data are available, it may not be possible to train a deep-learning model from scratch. An alternative approach is to fine-tune existing large language models (trained either on general or scientific texts) to a target task via transfer learning. This process reduces the need for labelled data as it uses fewer examples than would be needed to train a full language model. Through transfer learning a variety of NLP tasks could feasibly be performed by a single neural language model. Thus, LLMs allow the flexible development of custom pipelines without the specialized linguistic knowledge required to build traditional NLP tools. While there exist pre-trained language models designed to work with scientific literature, they are either explicitly biomedical focused, such as bioBERT [53] and PubMedBERT [54], or the majority of the training corpus is from the biomedical domain, e.g. scispaCy [14] and sciBERT [51]. The development of language models specifically for ecology and evolution, or disciplines that bridge ecology and biomedical sciences, such as veterinary medicine, could foreseeably be achieved by biomedical or more generalized language models as a foundation. A recent example is TaxoNERD [16], a model for taxonomic named entity recognition trained with transfer learning and updating of the bioBERT biomedical language model. While transfer learning offers a viable avenue for adapting LLMs for tasks in ecology and evolution, it requires the curation of labelled training data specific for each task.

### (c) Generative LLMs and prompt-based interaction for complex tasks

The training and fine-tuning of LLMs are computationally expensive tasks which rely on the availability of labelled training data. Developing domain-specific language models may be necessary for specialized tasks, but as language models are adapted to answer questions and respond directly to natural language prompts, it may be more efficient to directly prompt LLMs to identify, extract, and harmonize data directly from the scientific literature [57]. As an alternative to training domain-specific models, prompt-based learning with a pre-trained, general-purpose LLM allows for the execution of domain-specific tasks without the need for pre-training or transfer learning [72]. By additionally training LLMs to act as generative question answering models (e.g. OpenAI’s ChatGPT built on GPT, and Meta’s Llama-2) [73,74], researchers can directly prompt models to perform more complex information extraction tasks [75], as has been demonstrated for identifying potential pest controllers [57], hosts and pathogens [58], and the locations of species observations [76]. In this context, a ‘prompt’ refers to a piece of text to instruct a language model to perform a task in natural language rather than through computer code. Prompts may include explicit examples of the task to be completed (few-shot learning) or be composed of an instruction only (zero-shot learning). Prompt-based learning has become a popular area of research in recent years [77], with applications across multiple scientific disciplines, including text classification [77–79], NER [79,80] and relation extraction [78,81–85]. While obtaining reliable performance of LLMs on complex tasks is challenging and may require extensive experimentation, there is a rapidly growing repertoire of prompting techniques that show promise.

Least-to-most prompting [43] proposes to decompose a complex task into a series of subtasks, each as its own prompt, which are then queried in sequence. For example, in a task that involves both NER and RE, one could first prompt the LLM with an NER task (e.g. ‘list all species mentioned in the text’) and then detail the RE task and any other instructions (e.g. ‘return a table with two columns: if a listed species acts as a parasite of another species mentioned in the text [based on criteria *x*, *y*, *z*], then return this parasite in column 1 and its host(s) in column 2’) [57]. Least-to-most prompting has been shown to improve the ability of GPT-3 to solve difficult reasoning problems [43]. Similarly, chain-of-thought prompting uses a series of examples in the prompt to show intermediate reasoning, which aids the model in decision-making and can improve performance on a variety of reasoning tasks [82]. These techniques may be improved by allowing the model to sample multiple reasoning paths and generate a result based on the most consistent answer [83,84]. Such approaches are likely to be helpful in ecology to simultaneously extract entities and make decisions about their ecological roles (e.g. as pest or parasite) and relationships to other entities (e.g. host–parasite or prey–predator relations) [57,58]. While prompt-based interactions can be quite useful, the construction of an effective prompt may be highly task- and data-dependent, and thus may require extensive fine-tuning to account for intricacies, ambiguities, or exceptions. Some of the difficulties of effective prompt engineering may be circumvented by fine-tuning the LLM parameters through transfer learning, however, prompt-based approaches may offer a substantial reduction in computational cost if using existing LLMs.

## 3. Conclusion

NLP is a rapidly developing field which offers a diversity of approaches useful to researchers in ecology and evolution. Deep learning has revolutionized NLP approaches and now allows researchers without extensive linguistics or computational training to employ them. In particular, the proliferation of generative LLMs will likely foster a wide adoption of text mining in ecology, especially for complex tasks related to data extraction and synthesis of biodiversity data. However, given the energy and computational resources needed to train and implement deep learning-based language models, we emphasize that many of the simpler rule-based and traditional NLP approaches reviewed here may have comparable performance while requiring fewer resources. Currently, in ecology and evolution, our ability to develop state-of-the-art text mining tools is limited by the lack of open access to full texts of scientific publications [1]. Relevant to this is that university libraries are fundamentally changing; we now have ‘digital librarians’ and ecologists can advocate for this type of science to be supported through their libraries and contracts with publishers.

We are also in the midst of an exponential growth in the size and number of language models, both open and closed-source. With closed-source models such as OpenAI’s ChatGPT using subscription-based payments for access to the newest models and pay-per-token for legacy models, which may become unavailable with limited notice, the use of closed-source models or software reliant on them will create an unprecedented challenge for reproducible research [86]. Research code is already suffering from issues of reproducibility [87], and the adoption of closed-source models that lack transparency in their training data and/or model architecture directly opposes the open science movement. Competitive open-source LLMs are being released, but these do not mitigate the resource and environmental costs of LLMs (e.g. at least 400 GB of memory is needed to swiftly run the Falcon 180 billion parameter model [88]).

Prompt-based interaction with generative LLMs can increase the efficiency of data synthesis, but there is a risk that these models generate false or harmful information [41]. However, ecology and evolution have a rich history of literature-based data synthesis which can be leveraged for fine-tuning foundation models and validating outputs. Furthermore, the potential for LLMs to interact with external databases through retrieval-augmented generation (RAG) shows great promise for reducing false information [89] and building models that can keep up with rapidly expanding literature. Significant open questions for the future remain: how important is it to develop field-specific language models versus using general LLMs or transfer learning from foundation models from related disciplines? How can we ensure the tools we adopt will be open-access and developed using transparent methods? Should we attempt to limit the environmental impacts of training competing large-scale models, instead of using simpler approaches or transfer learning where possible? Regardless of how this methodological landscape develops, it will be important to keep the ‘human in the loop’, from sense-checking models and training data, identifying harmful or inaccurate outputs of generative models, balancing model complexity with environmental impacts, and ensuring the reproducible, open, and equitable access of literature, data, code, and models.

Table 1: Glossary of Key Terms and Concepts (alphabetical)

**Table 1.** Glossary of Key Terms and Concepts (alphabetical)

*Accuracy:* a common metric used to evaluate the performance of a machine learning model. Accuracy measures how often the model correctly predicts the outcomes within a given dataset. It is calculated by dividing the number of correct predictions by the total number predictions.
*Anaphora/cataphora:* a word or phrase used as a substitute for a word or phrase used earlier (anaphora) or later (cataphora) in a text. These are used to avoid repetition (e.g. it, they, their, some. For example, in ‘In the second week after hatching eagle chicks can hold up their heads’, ‘their’ is an anaphor, referring back to the eagle chicks.)
*Artificial neural network:* a type of machine learning model comprising a collection of connected units or nodes called artificial neurons organised into layers. Each connection between neurons has an associated weight. The process of training a neural network involves feeding it labelled training data, and adjusting the weights and biases during each iteration to minimise the difference between the predicted outputs and the true labels. This process is often done using optimization algorithms such as gradient descent.
*‘Bag of words’ (BOW) approaches:* methods that treat words (or n-grams) as independent entities, ignoring their common linguistic function (i.e. part of speech), or relative position in a sentence. Examples include word clouds, TF-IDF, and topic modelling.
*Bidirectional Encoder Representations from Transformers (BERT):* an open source machine learning framework developed by Google [33] based on the transformer deep learning architecture [34]. BERT models advanced previous neural language models by using self-attention mechanisms to understand the relationships between words in a sentence. BERT considers all words in a sentence at once, making it better at capturing context and meaning of given words. Also, by pre-training on large amounts of data rather than being trained from scratch, BERT is a general-purpose language model, allowing it to be fine-tuned for a wide variety of tasks.
*Corpus:* a collection of texts. Plural ‘corpora’ refers to multiple collections of texts.
*Data augmentation:* a technique commonly used in machine learning to improve the generalisation and robustness of a machine learning model. The idea is to increase the diversity of a training dataset by generating several slightly-modified copies of existing data.
*Deep learning:* a subset of machine learning based on artificial neural networks. Inspired by the human brain, these models use multiple layers of processing to analyse progressively more abstract higher level features from data. This allows deep learning models to recognize and simulate complex patterns in images, text, sound, and other types of data.
*Disambiguation:* the process of determining which sense of a word is meant in a given context. For example, ‘bark’ could refer to the bark of a tree, or the sound dogs make.
*Distant supervision:* a technique used in the field of machine learning, particularly in NLP and IE, which consists of using external knowledge bases to automatically label a large amount of data.
*Encoder-decoder neural network:* a type of deep neural network architecture that involves a two stage process. First input data are encoded into a numerical representation (typically a fixed length vector, often referred to as a hidden or latent representation). The decoder then takes this latent representation and produces an output in the desired format. This architecture is often used when the input is sequence data and the output is another type of sequence data. Different types of neural networks such as RNN, LSTM, CNN use this architecture.
*Feature engineering:* the process of using domain knowledge, curation, and data transformation to create predictor variables for predictive models.
*Few-shot learning:* a machine learning paradigm that focuses on training models with only a small number of examples for each class or task. The term ‘shot’ refers to the number of examples provided for each class during training. Extreme scenarios of few-shot learning include one-shot learning and zero-shot learning.
*Foundation model:* a large, pre-trained language model that is not trained for specific tasks but rather aims to learn general language representations that can be fine-tuned for specific downstream tasks with relatively small amounts of task-specific labelled data.
*Gold standard:* a set of data that have been manually curated and verified to be the most accurate or correct for a given analysis. In NLP the gold standard typically represents a set of documents that have been expertly annotated and against which different models can be trained or tested.
*Information retrieval (IR):* the process of searching for and identifying documents that are relevant for a given task.
*Language model:* a type of statistical or machine learning model designed to capture the inherent structure, patterns, and semantics of one or several human languages. A language model is generally trained to predict which word is more likely to appear next in a sentence based on the previous words.
*Large language model (LLM):* a deep neural network-based language model trained on massive amounts of text using self-supervision. The largeness of these models is a key factor in their ability to generalise well across a wide range of language-related tasks. GPT, the model behind the popular chatbot ChatGPT, is an example of a large language model.
*Latent Dirichlet Allocation (LDA):* a generative statistical model for the unsupervised classification of documents. One of the most popular methods for topic modelling, LDA aims to identify hidden topics that a document belongs to based on the words it contains.
*Lemma & Lemmatization:* a lemma is the canonical form of a word (e.g. the lemma of eats, eating, eaten, and ate is ‘eat’). Lemmatization is the process of determining the lemma of a word based on context and intended meaning.
*N-grams:* a set of N words that appear next to one another. For example, in the text ‘big oak tree’, ‘big’, ‘oak’, and ‘tree’ are unigrams, ‘big oak’ and ‘oak tree’ are bi-grams, and ‘big oak tree’ is a tri-gram.
*Natural Language Processing (NLP):* a subfield of computer sciences primarily concerned with giving computers the ability to understand, interpret, and generate human language.
*One-hot encoding:* a technique used to represent categorical variables as numerical values in a machine learning model. It converts categorical variables into vectors of binary values, where each category is represented by a unique index in the vector.
*One-shot learning:* an extreme case of a few-shot learning problem where a model is trained to recognize or classify new objects or patterns based on a single example.
*Ontology:* A semantic model of objects and their relationships in a domain of interest [90]. Ontologies formally define terms and concepts in a way that provides cross-references and semantic meaning.
*Out-of-vocabulary (OOV) words*: words that are not present in the vocabulary or training set of a model. These are words that the model has not encountered during its training phase and, as a result, may not be able to properly handle or understand when encountered in new, unseen data.
*Pipeline:* in NLP, a series of processing steps involved in the analysis and transformation of raw text into a structured format. Each step in the pipeline performs a specific NLP task, such as tokenization, part-of-speech tagging, named entity recognition, and the output of one step becomes the input for the next.
*Prompt:* a specific instruction or query given to a language model or system to perform a task or generate a response.
*Recommender system:* a type of software application or algorithm designed to provide personalised suggestions or recommendations to users. The goal of a recommender system is to predict and suggest items that users might be interested in, based on their preferences, historical behaviour, or the behaviour of similar users.
*Regular expression:* a pattern of rules that can be used to search, match, or alter text strings. Regular expressions are capable of capturing variation in capitalisation, number and type of characters, adjacency of particular characters, which characters start or end the text, and wildcards allowing any characters in specific positions.
*Self-attention:* a weighing mechanism used in deep learning models, particularly in the Transformer architecture, which allows the model to focus on relevant parts of the input sequence when making predictions or generating outputs.
*Sentence segmentation:* a fundamental NLP process, also known as sentence boundary detection, which involves breaking down a given text into individual sentences.
*Stemming:* a text normalisation technique used in NLP to reduce words to their base or root form, known as the ‘stem.’ For example, the words ‘ecology’, ‘ecological’ and ‘ecologist’ can all be reduced down to the common word stem ‘ecolog.’
*Stop words:* words which are commonly used in a language but which are generally considered to have little value in terms of meaning and are unlikely to contribute to the understanding of the content. These words are often filtered out to reduce the dimensionality of the data and focus on the most significant and informative words in a text.
*Supervised learning:* a machine learning paradigm in which a model is trained on a labelled dataset, i.e. a dataset where the input data (for example, a vector of predictor variables or an image) is paired with desired output labels or target values. The goal of supervised learning is to learn a mapping from input data to the corresponding output, enabling the model to make accurate predictions or classifications on new, unseen data.
*Surface form:* The form of a word as it appears in a text.
*Self-supervised learning:* a machine learning paradigm in which a model is trained using the data itself as a source of supervision, instead of relying on external labels provided by humans. This allows models to learn from large amounts of unlabelled data. Self-supervised learning has played a pivotal role in the emergence and success of large language models.
*Term:* a single word or a combination of words that convey a specific meaning. Terms are the building blocks of language, and in the context of NLP, they are often the units of interest when analysing and processing text.
*Term-frequency-inverse document frequency (tf-idf):* a numerical statistic used to evaluate the importance of a term (word) in a document relative to a collection of documents. The tf–idf is the product of two statistics, term frequency and inverse document frequency. Term frequency measures how often a term appears in a document. The inverse document frequency is a measure of how common or rare a term is across all documents. Higher tf-idf scores indicate that a term is both frequent within a specific document and rare across the entire corpus, making it more discriminative and potentially more important for representing the content of that document.
Tokenization & Tokens: Tokenization is the process of cutting up texts into small pieces (tokens) that can be processed by a computer. Historically text was segmented into ‘sentences’ and ‘words’ due to intuitive understanding of language and technical constraints. However, now more complex tokenization considering punctuation, word-forms, morphological derivatives, and sub-words are often used (see [10]) for a survey of tokenization approaches from initial conception to the deep-learning era).
*Topic modelling:* an NLP technique used to identify topics present in a corpus. The goal is to automatically discover latent topics that characterise the main themes or subjects within a collection of documents.
*Transformer:* a type of deep neural network architecture. Transformer architecture is particularly well known for its ability to efficiently capture long-term dependencies in sequential data using the self-attention mechanism, making it well suited to tasks involving sequential data. Transformers have proven to be highly efficient and scalable, leading to the development of large pre-trained transformer models that have had a significant impact on various NLP tasks and beyond.
*Unstructured data:* any data that does not have a predefined data model or organisation. Unlike structured data, which is typically organised in rows and columns (e.g., relational databases), unstructured data lacks a clear and organised structure. Examples of unstructured data include text, multimedia data, web pages, sensor data, etc.
*Unsupervised learning:* a machine learning paradigm in which a model is trained on unlabeled data, and the goal is to discover the inherent structure or patterns present in the data without explicit supervision in the form of labelled outcomes.
*Vector representation:* the encoding of objects, such as words or documents, as vectors in a multidimensional space. Vector representations are used to represent the semantic meaning and relationships between objects in a way that can be leveraged by machine learning models. Two common types of vector representations are one-hot encoding and word embeddings.
*Weak supervision:* a scenario in machine learning where the training data is labelled with noisy, limited, or imprecise annotations instead of high-quality, fully accurate labels.
*Word cloud:* a visual depiction of the common words in a document, often with the size of each word in proportion to its relative frequency.
*Word embedding:* a dense vector representation of words in a continuous vector space such that words with similar meanings are closer to each other in the vector space. Word embeddings are classically learned or obtained from pre-trained language models and used as features in various NLP tasks.
*Word sense:* the various meanings or interpretations that a word can have in different contexts.

## Data Availability

This article has no additional data.
